# Immersed isogeometric analysis with boundary conformal quadrature for finite deformation elasticity

**DOI:** 10.1007/s00419-025-02924-2

**Published:** 2025-08-31

**Authors:** Yusuf T. Elbadry, Pablo Antolín, Oliver Weeger

**Affiliations:** 1 Cyber-Physical Simulation, Department of Mechanical Engineering and Graduate School Computational Engineering, Technical University of Darmstadt, Dolivostr. 15, 64293 Darmstadt, Germany; 2 Institute of Mathematics, École Polytechnique Fédérale de Lausanne, 1015 Lausanne, Switzerland

**Keywords:** Fictitious domain method, Immersed isogeometric analysis, Finite deformation, Hyperelastic material, Boundary conformal quadrature

## Abstract

Numerical simulation of complex geometries can be an expensive and time-consuming undertaking, in particular due to the lengthy preparation of geometry for meshing and the meshing process itself. To tackle this problem, immersed boundary and fictitious domain methods rely on embedding the physical domain into a Cartesian grid of finite elements and resolving the geometry only by adaptive numerical integration schemes. However, the accuracy, robustness, and efficiency of immersed or cut cell approaches depends crucially on the integration technique applied on trimmed cells. This issue becomes more apparent in nonlinear problems, where intermediate solution steps are necessary to achieve convergence. In this work, we adopt an innovative algorithm for boundary conformal quadrature that relies on a high-order B-spline re-parameterization of trimmed elements to address small and large deformation elasticity problems. We accomplish this using spline-based immersed isogeometric analysis, which eliminates the need for body conformal finite element mesh. The integration points are obtained by applying classical Gauss quadrature to conformal re-parameterizations of the cut elements, whereas the discretization itself is not refined. This ensures a precise integration with minimum quadrature points and degrees of freedom. The proposed immersed isogeometric analysis with boundary conformal quadrature is evaluated on benchmark problems for 2D linear and nonlinear elasticity. The results show convergence with optimal rates in *h*-and *k*-refinement, thus demonstrating the efficiency and the precision of the method. As demonstrated, in conjunction with the simple to implement penalization and deformation map resetting approaches in the fictitious domain, it performs robustly also for finite deformations. Furthermore, it is exemplified that the method can be easily applied for multiscale homogenization of microstructured materials in the large deformation regime.

## Introduction

The finite element method (FEM) is a well-established numerical technique for solving boundary value problems of partial differential equations (PDEs) and associated boundary conditions. These PDEs encapsulate the dynamics of real systems and processes, often characterized by the presence of nonlinearities [93]. The importance of using simulations for such systems lies in providing an alternative way of reducing the time and cost associated with experimental endeavors. This simulation approach finds application in various fields, including optimization efforts [25, 85, 87], the generation of data for multiscale constitutive modeling using artificial neural networks [41–43, 51], and the numerical extension and comparison of existing material models with experimental results [6, 84, 91].

**Fig. 1:**
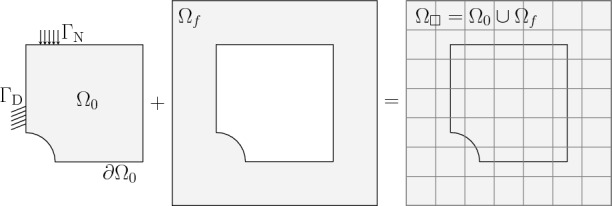
Schematic drawing for the immersed boundary concept, where the extended domain $\Omega _\square$ represents the union of the physical domain $\Omega _0$ , which is described by its boundary representation $\partial \Omega _0=\Gamma _\textrm{D}\cup \Gamma _\textrm{N}$, and the fictitious domain $\Omega _f$

The core idea of the finite element method (FEM) is to discretize the domain into a set of standardized elements, forming what is commonly known as a mesh, in order to accurately resolve the domain boundaries and provide a numerical discretization of the sought field variables, e.g., displacements in elasticity. While this step may appear straightforward, it becomes intricate and time-consuming, particularly when dealing with structures characterized by complex geometries. In this regard, the introduction of isogeometric analysis (IGA) [13, 38] marked a critical advancement in bridging computer-aided design (CAD) and finite element simulations. IGA achieves this by representing both the geometry and the solution field through B-splines or Non-Uniform Rational B-splines (NURBS) [67]. While IGA contributes to addressing accuracy concerns by precisely representing geometries, the challenge of creating boundary-fitted meshes persists for intricate geometries. Consequently, automating (isogeometric) finite element simulations necessitates an efficient technique capable of accurately resolving complex geometries, e.g., through multi-patch discretizations [64] or boundary conformal trimming [79].

Immersed boundary methods (IBM) were introduced as a solution to obviate the generation of boundary conforming meshes [65]. The fundamental concept underlying these methods involves immersing the physical domain into an extended domain, conceptualized as the union of the actual domain and a fictitious domain, as illustrated in Fig. 1. Numerous approaches to “immersed boundary” or “fictitious domain” methods have been proposed in the literature, including marker and cell [28, 31], volume of fluid [59, 68], level-set [62], cut cell [50], and fictitious domain methods, alongside penalty methods [8, 69], the extended finite element method [7, 27, 78, 82], the CutFEM method [11], and the finite cell method (FCM) [63].

A major concern when using the aforementioned methods is the accurate integration over trimmed elements. Methods therefore can be primarily classified into three categories:

- Methods that reduce the dimension of the integral by applying the divergence theorem [1, 30, 83]. However, finding the antiderivative for the integrand is not straightforward. To overcome this, the integrand must either be assumed to have a certain form [12] or approximations must be obtained [30].
- Moment fitting methods, where the locations and weights of the integration points are determined by solving a local system of equations [29, 57, 81, 89]. A significant advantage of moment fitting is the retention of the minimum number of integration points. Initially, these methods produced negative weights, which led to stability issues. However, non-negative moment fitting methods were introduced to overcome this problem [47, 48]. Additionally, they need to be complemented with other methods to accurately compute the right-hand side vector.
- Decomposition-based methods, which partition a complex domain into simple cells where standard quadrature rules can be directly applied. These methods can be categorized into low-order and high-order methods. An example of a low-order method is quad- or octree subdivision [63, 75], which unfortunately requires a large number of integration points to accurately resolve the geometry. In contrast, high-order decomposition methods provide a high-order approximation to the trimmed geometries and require significantly fewer integration points compared to adaptive refinement methods. This high-order decomposition is ideally suited for implementation within the IGA framework. Furthermore, these methods involve triangulations or complex partitioning [2, 53], which generally require a positive Jacobian. However, this is not necessary for integration purposes as shown in [4].

In solid mechanics, the FCM has been widely employed, in which material parameters within the fictitious domain are penalized to eliminate its effects. In the early stages of developing the FCM, the approach was combined with hierarchical quadtree/octree refinement to ensure accurate integration along the boundaries of the physical domain. Trimmed elements were subdivided into equal cells, with each trimmed subcell undergoing further refinement until a specified level was reached [19, 63, 74, 77]. For each subcell, the Gauss quadrature rule [37] was applied, thereby facilitating the integration process. The FCM has found application in various domains, including solving linear elasticity [63], addressing problems related to thin-walled structures [71], handling linear thermoelasticity [90], material homogenization for intricate microstructures [20], and addressing large deformation problems [77]. Further details on the FCM and its application can be found in [75].

While the use of immersed domain methods, such as the FCM, has successfully mitigated meshing challenges, numerical integration remains a concern due to the substantial number of Gauss points required in quadtree/octree schemes, leading to extended computational times and post-processing efforts [17]. Some strategies have been proposed to mitigate such computational burden, e.g., [44, 66]. Among them, moment fitting technique variants were introduced to reduce the number of Gauss points needed for accurate integrals [36, 40, 56]. However, their lower stability compared to adaptive integration prompted the development of a new adaptive scheme based on moment fitting for nonlinear problems [35]. As a result, a non-negative moment fitting quadrature method was introduced to address stability issues, significantly reducing the required number of Gauss points for both linear elastic and small strain elastoplastic problems [47], as well as for simulations involving large deformations [26]. In pursuit of a robust and accurate integration technique, the immersed boundary conformal (IBC) isogeometric analysis method was introduced as a substitute for adaptive integration schemes for two-dimensional problems [86]. Additionally, a decomposition-based method was proposed to accurately represent and resolve three-dimensional geometries [4]. This re-parameterization approach is somewhat similar to boundary conformal isogeometric methods for trimmed geometries, see, e.g., [79], however, in the immersed setting only adapted quadrature points are obtained and the discretization itself is not refined or adapted to the boundary. Another similar approach can be found in [48], however it is not applicable to the IGA framework.

In this work, the isogeometric IBC concept is transferred to small and large deformation elasticity, focusing on two-dimensional problems. In this context, it is demonstrated that the approach can deliver highly accurate and robust numerical solutions for nonlinear problems in an efficient manner. The further outline of this manuscript is as follows. The basics of continuum mechanics and small and finite deformation elasticity are introduced in Sect. 2. Then, in Sect. 3, the proposed immersed isogeometric finite element concept is outlined, including the boundary conformal quadrature approach, deformation map resetting to enhance stability of the nonlinear solution process, as well as weak enforcement of essential boundary conditions through penalty and Nitsche’s method. This approach is numerically evaluated and applied in Sect. 4, using benchmark problems for linear and nonlinear elasticity, as well as an example for numerical homogenization of microstructures. Finally, Sect. 5 concludes the paper and suggests directions for future research.

## Finite deformation elasticity

In this section, we summarize the basic continuum mechanical background needed to describe the behavior of hyperelastic materials [34]. The first subsection introduces finite deformation kinematics and hyperelastic constitutive models. This is followed by formulating the strong and the weak forms of finite strain elasto-statics in the reference and current configurations. Finally, we also present the restriction to linear elasticity at infinitesimal strains.

### Kinematics and constitutive model

To describe the motion of a material particle in a *d*-dimensional body $\Omega _0\subset {\mathbb {R}}^d$ ($d=2,3$) from the reference configuration ${{\textbf {X}}}\in \Omega _0$ to the current configuration ${{\textbf {x}}}\in \Omega \subset {\mathbb {R}}^d$, we use the deformation map $\varvec{\varphi }:\Omega _0\rightarrow \Omega$ defined as

1 $$\begin{aligned} {{\textbf {x}}}= \varvec{\varphi }({{\textbf {X}}}). \end{aligned}$$

The displacement field ${{\textbf {u}}}:\Omega _0\rightarrow {\mathbb {R}}^d$ is the difference between the current and the reference position vectors

2 $$\begin{aligned} {{\textbf {u}}}= \varvec{\varphi }({{\textbf {X}}}) - {{\textbf {X}}}. \end{aligned}$$

The deformation gradient tensor ${\textbf{F}}$ links a material line element in the reference configuration $\text {d}{\textbf{X}}$ to a material line in the current configuration $\text {d}{\textbf{x}}$ as following

3 $$\begin{aligned} \text {d}{\textbf{x}} = {\textbf{F}}\ \text {d}{\textbf{X}}. \end{aligned}$$

Thus, it can be defined as the gradient of the deformation map $\varvec{\varphi }({{\textbf {X}}})$ as

4 $$\begin{aligned} {\textbf{F}} = \frac{\partial {{\textbf {x}}}}{\partial {{\textbf {X}}}} = \nabla _{{{\textbf {X}}}} \varvec{\varphi }({{\textbf {X}}}) = {\textbf{I}} + \nabla _{{{\textbf {X}}}} {{\textbf {u}}}\end{aligned}$$

A volume element in the reference configuration $\text {d}V$ is linked to its counterpart in the current configuration $\text {d}v$ through the Jacobian *J*, i.e., the determinant of the deformation gradient, as

5 $$\begin{aligned} \text {d}v = J\ \text {d}V, \qquad J = \textrm{det}({\textbf{F}}). \end{aligned}$$

Based on the deformation gradient, the right Cauchy-Green tensor ${\textbf{C}}$ and the Green–Lagrange strain tensor ${\textbf{E}}$ can be defined as objective strain measures as:

6 $$\begin{aligned} \begin{aligned} {\textbf{C}}({\textbf{X}})&= {\textbf{F}}^{\top }{\textbf{F}},\\ {\textbf{E}}({\textbf{X}})&= \frac{1}{2}({\textbf{C}}({\textbf{X}}) - {\textbf{I}}). \end{aligned} \end{aligned}$$

A constitutive model relates the strain measures to stresses. For hyperelastic materials, material models are defined in terms of a free energy function $\Psi$:

7 $$\begin{aligned} \Psi ({{\textbf {F}}}) = \Psi ({{\textbf {E}}}) = \Psi ({{\textbf {C}}}) = \Psi (I_1,I_2,I_3), \end{aligned}$$

where the invariants of the right Cauchy–Green tensor, which can be employed for isotropic materials, are defined as

8 $$\begin{aligned} \begin{aligned} I_1&= \textrm{tr}({{\textbf {C}}}), \quad I_2 = \frac{1}{2} \left( \textrm{tr}({{\textbf {C}}})^2 - \textrm{tr}({{\textbf {C}}}^2) \right) , \quad I_3 = \textrm{det}({{\textbf {C}}})=J^2. \end{aligned} \end{aligned}$$

Then, the first Piola–Kirchhoff stress tensor ${{\textbf {P}}}$, the second Piola–Kirchhoff stress ${{\textbf {S}}}={{\textbf {F}}}^{-1}{{\textbf {P}}}$ and the elasticity tensor ${\mathbb {C}}$ can be obtained by differentiating the free energy function $\Psi$ as

9 $$\begin{aligned} \begin{aligned} {{\textbf {P}}}&= \frac{\partial \Psi ({{\textbf {F}}})}{\partial {{\textbf {F}}}},\\ {{\textbf {S}}}&= \frac{\partial \Psi ({{\textbf {E}}})}{\partial {{\textbf {E}}}}= 2\frac{\partial \Psi ({{\textbf {C}}})}{\partial {{\textbf {C}}}},\\ {\mathbb {C}}&= \frac{\partial {{\textbf {S}}}}{\partial {{\textbf {E}}}} = \frac{\partial ^2 \Psi ({{\textbf {E}}})}{\partial {{\textbf {E}}}\, \partial {{\textbf {E}}}} = 4\frac{\partial ^2 \Psi ({{\textbf {C}}})}{\partial {{\textbf {C}}}\, \partial {{\textbf {C}}}}. \end{aligned} \end{aligned}$$

Here, for the sake of exposition, we adopt a Mooney–Rivlin-type material model for incompressible 2D elasticity under plane stress conditions, which is given by the strain energy function

10 $$\begin{aligned} \Psi (I_1,I_2)= A_{10}(I_1 - 3) + A_{01}(I_2 - 3) \end{aligned}$$

with parameters $$A_{01},A_{10}>0$$.

### Strong and weak forms of boundary value problem

For a body under static loads, the conservation of linear momentum written in the reference configuration reads

11 $$\begin{aligned} \nabla _{{{\textbf {X}}}}\cdot {{\textbf {P}}}+ \rho _0\,{{\textbf {b}}}= 0 \quad \forall {{\textbf {X}}}\in \Omega _0, \end{aligned}$$

where $\rho _0$ is the initial mass density and ${{\textbf {b}}}$ is the body weight force. To complete the boundary value problem for elasto-statics, the following Dirichlet and Neumann boundary conditions are needed

12 $$\begin{aligned} \begin{aligned} {{\textbf {u}}}&= \bar{{{\textbf {u}}}}\  &   \text {on}\ \ \Gamma _\textrm{D},\\ {{\textbf {P}}}\ {{\textbf {n}}}&= {{\textbf {t}}}\    &   \text {on}\ \ \Gamma _\textrm{N}, \end{aligned} \end{aligned}$$

where $\Gamma _\textrm{D},\Gamma _\textrm{N}\subset \partial \Omega _0$, such that $\Gamma _\textrm{D}\cup \Gamma _\textrm{N}=\partial \Omega _0$ and $\Gamma _\textrm{D}\cap \Gamma _\textrm{N}=\emptyset$, represent the domain’s boundaries, where either a displacement $\bar{{{\textbf {u}}}}$ or a traction ${{\textbf {t}}}$ are prescribed, and ${{\textbf {n}}}$ is the unit normal vector on the boundary in the outward direction.

Through the methods of weighted residuals, or alternative also by the principle of virtual work or minimum of potential energy, the weak or variational form of the balance equation Eq. (11) can be derived as

13 $$\begin{aligned} \begin{aligned} \delta {\mathcal {W}}({{\textbf {u}}},\delta {{\textbf {u}}})&= \delta {\mathcal {W}}_{\text {int}}({{\textbf {u}}},\delta {{\textbf {u}}}) - \delta {\mathcal {W}}_{\text {ext}}(\delta {{\textbf {u}}})= 0 \quad \forall \delta {{\textbf {u}}}, \end{aligned} \end{aligned}$$

with variations of internal and external work as

14 $$\begin{aligned} \begin{aligned} \delta {\mathcal {W}}_{\text {int}}({{\textbf {u}}},\delta {{\textbf {u}}}) =&\int _{\Omega _0} {{\textbf {P}}}: \nabla _{{{\textbf {X}}}} \delta {{\textbf {u}}}\ \text {d}V =\int _{\Omega _0} {{\textbf {S}}}: \delta {{\textbf {E}}}\ \text {d}V ,\\ \delta {\mathcal {W}}_{\text {ext}}(\delta {{\textbf {u}}}) =&\int _{\Omega _0} \rho _0\ {{\textbf {b}}}\cdot \delta {{\textbf {u}}}\ \text {d}V +\int _{\Gamma _\textrm{N}} {{\textbf {t}}}\cdot \delta {{\textbf {u}}}\ \text {d}A, \end{aligned} \end{aligned}$$

where $$\delta {{{\textbf {E}}}} = \frac{1}{2} ( (\nabla _{{\textbf {X}}}\delta {{\textbf {u}}})^\top {{\textbf {F}}}+ {{\textbf {F}}}^\top \nabla _{{\textbf {X}}}\delta {{\textbf {u}}})$$.

Due to the nonlinearity of Eq. (13), its numerical solution, c.f. Sect. 3.3, requires the linearization around a known state ${{\textbf {u}}}$ using Taylor series, see [88] . We take the derivative in the direction of the incremental displacement $\Delta {{\textbf {u}}}$ and hence the linearized weak form $\text {D}\delta {\mathcal {W}}$ consists of two parts, a geometrical part $\text {D}\delta {\mathcal {W}}^{\text {geo}}$ and a material part $\text {D}\delta {\mathcal {W}}^{\text {mat}}$ as

15 $$\begin{aligned} \begin{aligned} \text {D}\delta {\mathcal {W}}({{\textbf {u}}},\delta {{\textbf {u}}})\cdot \Delta {{\textbf {u}}}&= \int _{\Omega _0} \nabla _{{\textbf {X}}}\Delta {{\textbf {u}}}\cdot {{{\textbf {S}}}}\cdot \nabla _{{\textbf {X}}}\delta {\varvec{u}}\ \text {d}V + \int _{\Omega _0} \delta {{{\textbf {E}}}} : {{\mathbb {C}}} : \Delta {{{\textbf {E}}}} \ \text {d}V \\&= \text {D}\delta {\mathcal {W}}^{\text {geo}} \cdot \Delta {{\textbf {u}}}+ \text {D}\delta {\mathcal {W}}^{\text {mat}} \cdot \Delta {{\textbf {u}}}, \end{aligned} \end{aligned}$$

where $$\Delta {{\textbf {E}}}={{\textbf {F}}}^\top \Delta {{\textbf {u}}}\,{{\textbf {F}}}$$.

### Linear elasticity

In the case of small deformations and strains, and linear elastic material behavior, the elasticity problem can be simplified.

Introducing the linear strain tensor $\varvec{\varepsilon }\,\dot{=}\,{{\textbf {E}}}$ as

16 $$\begin{aligned} \varvec{\varepsilon }({{\textbf {u}}}) = \frac{1}{2}\left( \nabla _{{{\textbf {X}}}}{{\textbf {u}}}+ (\nabla _{{{\textbf {X}}}}{{\textbf {u}}})^\top \right) , \end{aligned}$$

and assuming linear, isotropic material behavior, the Cauchy stress tensor can be expressed as

17 $$\begin{aligned} \varvec{\sigma }= \lambda \, \textrm{tr}(\varvec{\varepsilon })\, {{\textbf {I}}}+ 2 \mu \, \varvec{\varepsilon }= {\mathbb {C}}: \varvec{\varepsilon }({{\textbf {u}}}), \end{aligned}$$

where the Lamé parameters $\lambda$ and $\mu$ are defined in terms of the Young’s modulus *E* and Poisson’s ratio $\nu$ as

18 $$\begin{aligned} \lambda = \frac{E\ \nu }{(1+\nu )(1-2\nu )}, \quad \mu =\frac{E}{1+\nu }. \end{aligned}$$

Then, the weak form in Eq. (13) can be expressed in terms of the variation of internal work as

19 $$\begin{aligned} \begin{aligned} \delta {\mathcal {W}}^{\text {lin}}_{\text {int}}({{\textbf {u}}},\delta {{\textbf {u}}}) =&\int _{\Omega _0} \varvec{\varepsilon }({{\textbf {u}}}):{\mathbb {C}}:\varvec{\varepsilon }(\delta {{\textbf {u}}})\ \text {d}V . \end{aligned} \end{aligned}$$

## Immersed isogeometric analysis

The basic idea of isogeometric analysis is to represent not only the geometry by spline function such as B-splines and NURBS, as in computer-aided design, but also the numerical discretization of the field variables [38]. Though IGA was initially aimed at reducing or even eliminating the efforts of finite element meshing, it has been shown that the higher order and smoothness of basis functions is also beneficial in the context of fictitious domain methods, increasing accuracy and robustness [74, 76–78].

### B-splines

The building blocks of (immersed) IGA are B-splines [38, 67]. Given a knot vector $\{\xi _1,\ldots ,\xi _{n+p+1}\}$ that defines a parameter domain $\Omega _\xi =[\xi _1,\xi _{n+p+1}]$, the Cox-de Boor recursion formula can be used to define the *n* B-spline basis functions $N_{i,p}:\Omega _\xi \rightarrow {\mathbb {R}}$ of degree $p\ge 0$ as

20 $$\begin{aligned} \begin{aligned} N_{i,0}(\xi ) =&{\left\{ \begin{array}{ll} 1, &  \text {if } \ \xi _i \leqslant \xi < \xi _{i+1}\\ 0, &  \text {otherwise} \end{array}\right. },\\ N_{i,p}(\xi ) =&\frac{\xi - \xi _i}{\xi _{i+p} - \xi _i} N_{i,p-1}(\xi )+ \frac{\xi _{i+p+1} - \xi }{\xi _{i+p+1} - \xi _{i+1}} N_{i+1,p-1}(\xi ). \end{aligned} \end{aligned}$$

These basis functions are non-negative, linearly independent, and they satisfy the partition of unity property. Moreover, they offer controllable smoothness and can be up to $(p-1)$-times continuously differentiable at inner knots of multiplicity 1. Typically, open knot vectors are used, for which the first and last knot are repeated $(p+1)$-times, which makes the basis functions interpolatory at the boundaries.

Bivariate B-splines basis functions $N_i(\varvec{\xi })$ with $\varvec{\xi }=(\xi , \eta )$ can be constructed by the tensor product of two univariate B-spline basis functions as

21 $$\begin{aligned} \begin{aligned} N_i(\xi , \eta )&= N_{j,p}(\xi ) \cdot N_{k,q}(\eta ), \text {for}\quad i =(j-1)l+k \text { with}\left\{ \begin{array}{l} j=1,\ldots ,l,\\ k=1,\ldots ,m \end{array}\right. . \end{aligned} \end{aligned}$$

Here, $N_{j,p}:\Omega _\xi \rightarrow {\mathbb {R}}$ and $N_{k,q}:\Omega _\eta \rightarrow {\mathbb {R}}$ are the *l* and *m* univariate B-spline basis functions of degree *p* and *q*, respectively, as defined in Eq. (20). The $n=l\cdot m$ bivariate B-splines basis functions $N_i(\varvec{\xi }):{{\hat{\Omega }}}\rightarrow {\mathbb {R}}$ are then defined on the two-dimensional parameter domain ${{\hat{\Omega }}}=\Omega _\xi \times \Omega _\eta$. For simplicity of the notation, $i=1,\ldots ,n$ is used as a combined index and the dependence of $N_i$ on the degrees *p* and *q* is dropped. In the following, for the sake of clarity, we always set $p=q$ and $l=m$.

### Isogeometric discretization

A two-dimensional B-spline surface $\Omega _\square \subset {\mathbb {R}}^2$ can then be parameterized by ${{\textbf {X}}}^h:{{\hat{\Omega }}}\rightarrow \Omega _\square$ as a linear combination of *n* bivariate B-spline basis functions $N_i(\xi , \eta )$ with control points ${{\textbf {X}}}_i\in {\mathbb {R}}^2$:

22 $$\begin{aligned} {{\textbf {X}}}^h(\varvec{\xi }) = \sum _{i=1}^{n} N_{i}(\varvec{\xi })\, {{\textbf {X}}}_i. \end{aligned}$$

Within the framework of isogeometric analysis, B-spline surface parameterizations are not only used to describe the geometry in terms of the position vector ${{\textbf {X}}}^h$, but also to discretize and approximate the field variables:

23 $$\begin{aligned} \begin{aligned} {{\textbf {u}}}\approx {{\textbf {u}}}^h(\varvec{\xi })&= \sum _{i=1}^{n} N_{i}(\varvec{\xi })\, {{\textbf {u}}}_i, \\ \delta {{\textbf {u}}}\approx \delta {{\textbf {u}}}^h(\varvec{\xi })&= \sum _{i=1}^{n} N_{i}(\varvec{\xi })\, \delta {{\textbf {u}}}_i. \end{aligned} \end{aligned}$$

Here, ${{\textbf {u}}}_i,\delta {{\textbf {u}}}_i\in {\mathbb {R}}^2$ are the *n* control points of the discretized displacement field ${{\textbf {u}}}^h:{{\hat{\Omega }}}\rightarrow {\mathbb {R}}^2$ and the virtual displacement field $\delta {{\textbf {u}}}^h:{{\hat{\Omega }}}\rightarrow {\mathbb {R}}^2$, respectively.

Note that, similar to isoparametric finite elements, Eq. (23) defines both fields on the parameter domain ${{\hat{\Omega }}}$ and thus the inverse of the position vector field Eq. (22) has to be invoked to represent them on the physical domain $\Omega _\square$, i.e., ${{\textbf {u}}}^h({{\textbf {X}}})={{\textbf {u}}}^h(\varvec{\xi })\circ {{\textbf {X}}}^h(\varvec{\xi })^{-1}$. Thus, gradients w.r.t. the reference coordinates need to be computed using the chain rule, e.g.,

24 $$\begin{aligned} \nabla _{{{\textbf {X}}}} {{\textbf {u}}}^h(\varvec{\xi }) = \nabla _{\varvec{\xi }} {{\textbf {u}}}^h(\varvec{\xi }) \cdot (\nabla _{\varvec{\xi }} {{\textbf {X}}}^h(\varvec{\xi }))^{-1}, \end{aligned}$$

where $\nabla _{\varvec{\xi }} {{\textbf {X}}}^h(\varvec{\xi })=:{{\textbf {J}}}(\varvec{\xi })$ is the Jacobian of the geometric parameterization.

### Isogeometric finite elements

Substituting the discretizations from Eq. (23) into the weak form of nonlinear elasticity as given in Eq. (13) then leads to the $N=2n$-dimensional nonlinear system of equations25with the internal and external force vectors as

26 $$\begin{aligned} \begin{aligned} {{\textbf {f}}}_{\text {int},i}({{\textbf {u}}}^h)&= \int _{\Omega _\square } \nabla _{{{\textbf {X}}}} N_i^\top \, {{\textbf {P}}}({{\textbf {u}}}^h) \ \text {d}V= \int _{\Omega _\square } {{\textbf {B}}}_i({{\textbf {u}}}^h)^\top \, {{\textbf {S}}}({{\textbf {u}}}^h) \ \text {d}V, \\ {{\textbf {f}}}_{\text {ext},i}&= \int _{\Omega _\square } N_i\, {{\textbf {b}}}\ \text {d}V + \int _{\Gamma _\textrm{N}} N_i\, {{\textbf {t}}}\ \text {d}A. \end{aligned} \end{aligned}$$

Here, the operator ${{\textbf {B}}}_i$ is used to express the variation of the Green–Lagrange strain tensor w.r.t. $\delta {{\textbf {u}}}_i$ as:

27 $$\begin{aligned} \begin{aligned} {{{\textbf {E}}}}(\delta {{\textbf {u}}}_i)&= {{\textbf {B}}}_i({{\textbf {u}}}^h)\, \delta {{\textbf {u}}}_i= \frac{1}{2} \left( \nabla _{{\textbf {X}}}N_i^\top {{\textbf {F}}}({{\textbf {u}}}^h) + {{\textbf {F}}}({{\textbf {u}}}^h)^\top \nabla _{{\textbf {X}}}N_i\right) \delta {{\textbf {u}}}_i. \end{aligned} \end{aligned}$$

For the numerical integration of the integrals in the discretized weak form (25), it has to be recognized that B-splines are piecewise polynomials. Thus, we define $n_e$ disjunct elements ${{\hat{\Omega }}}^e\subset {{\hat{\Omega }}}$, $\bigcup _{e=1}^{n_e}{{\hat{\Omega }}}^e={{\hat{\Omega }}}$ by the knot spans of the knot vectors $\{\xi _1,\dots ,\xi _{l+p+1}\}\times \{\eta _1,\dots ,\eta _{m+q+1}\}$, e.g., ${{\hat{\Omega }}}^e=[\xi _j,\xi _{j+1}]\times [\eta _k,\eta _{k+1}]$. The images of the elements in the parameter domain are then denoted as $\Omega ^e={{\textbf {X}}}^h({{\hat{\Omega }}}^e)$. Then, the integration over the physical domain $\Omega _\square$ can be pulled-back to ${{\hat{\Omega }}}$ and be executed on the elements in the parameter domain ${\hat{\Omega }}^e$. Hence, same like in isoparametric FE, a standard Gauss quadrature rule with $n_{qp}=(p+1)\times (q+1)$ quadrature points is applied to each element [37, 38]. We numerically compute the basis functions, their gradients and the determinant of the pullback Jacobians at each integration point in the bi-unit parent element.

Applying the isogeometric discretization to Eq. (25), the integrals can be expressed as the summation of the integration over all the elements, e.g., for the internal force vector as

28 $$\begin{aligned} \begin{aligned} {{\textbf {f}}}_{\text {int},i}({{\textbf {u}}}^h)&=\int _{\Omega _0} {{\textbf {B}}}_i({{\textbf {u}}}^h)^\top \, {{\textbf {S}}}({{\textbf {u}}}^h) \ \text {d}V = \int _{{\hat{\Omega }}} {{\textbf {B}}}_i({{\textbf {u}}}^h)^\top \, {{\textbf {S}}}({{\textbf {u}}}^h) \ \det \,{{\textbf {J}}}(\varvec{\xi }) \ \text {d}{\hat{V}} \\&=\sum _{e=1}^{n_e} \int _{{\hat{\Omega }}^e} {{\textbf {B}}}_i({{\textbf {u}}}^h)^\top \, {{\textbf {S}}}({{\textbf {u}}}^h) \ \det \,{{\textbf {J}}}({\varvec{\xi }}) \ \text {d}{\hat{V}}\approx \sum _{e=1}^{n_e} \sum _{r=1}^{n_{qp}} w_r^e\, {{\textbf {B}}}_i(\varvec{\xi }_r^e)^\top \, {{\textbf {S}}}(\varvec{\xi }_r^e) \ \det \,{{\textbf {J}}}({\varvec{\xi }_r^e}), \end{aligned} \end{aligned}$$

where $w_r^e$ are the weights and $\varvec{\xi }_r^e$ the points of a standard Gauss quadrature rule on the parameter element ${{\hat{\Omega }}}^e$.

Once also the Dirichlet boundary conditions from Eq. (12) are incorporated, see Sect. 3.7, Eq. (25) can be solved for the $N=2 n$ unknown degrees of freedom, i.e., the *n* two-dimensional displacement control points, which are summarized in the vector . This nonlinear system of equations is typically solved incrementally for  using a Newton–Raphson method, where the vector of control point displacement increments  is obtained from the solution of the linear system:29Here, the tangential stiffness matrix  resulting from the linearized weak form Eq. (15) is obtained as:30with

31 $$\begin{aligned} \begin{aligned} {{\textbf {K}}}_{ij}^{\text {geo}}&= \int _{\Omega _\square } \nabla N_i^\top \, {{\textbf {S}}}({{\textbf {u}}}^h) \ \nabla N_j \ \text {d}V ,\\ {{\textbf {K}}}_{ij}^{\text {mat}}&= \int _{\Omega _\square } {{\textbf {B}}}_i({{\textbf {u}}}^h)^\top {\mathbb {C}}({{\textbf {u}}}^h)\ {{\textbf {B}}}_j({{\textbf {u}}}^h)\ \text {d}V , \end{aligned} \end{aligned}$$

where the integrals are computed over the elements in the parametric domain using Gauss quadrature, analogous to Eq. (28). The convergence of the solution is determined by the 2-norm of the residual  and the relative 2-norm of the displacement update .

The discretization of linear elasticity, c.f. Sect. 2.3, is analogous and corresponds essentially to solving Eq. (29) for  with , i.e., with right-hand side .

**Fig. 2:**
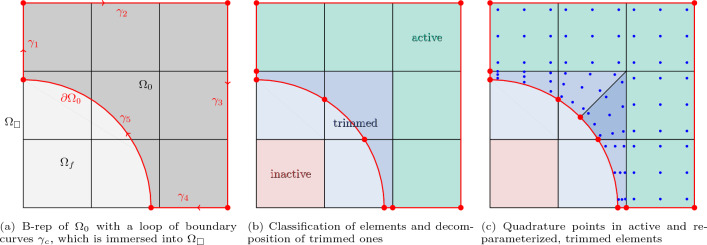
Schematic drawing of the concept of immersed IGA with boundary conformal quadrature. **a** The physical domain $\Omega _0$ (in dark gray) is immersed into an extended domain $\Omega _\square$, which consists of $3\times 3$ elements. **b** Active elements in green, trimmed elements in blue and non-active elements in red. **c** The integration points for the active parts of the trimmed and re-parametrized elements are shown in blue

### Immersed IGA

In standard isogeometric finite element analysis, the domain $\Omega _0$ is parameterized through a B-spline surface as stated in Eq. (22), i.e., $\Omega _0=\Omega _\square$. However, for such a bijective mapping ${{\hat{\Omega }}}\rightarrow \Omega _0$ to exist, $\Omega _0$ must be a topological square. For practical engineering problems, this is usually not the case and thus IGA requires the decomposition of the domain into a multi-patch geometry consisting of several topological squares, which is a cumbersome task-similar to finite element meshing [64].

Instead, the idea of immersed methods is to choose a very simple extended domain $\Omega _\square$ for the definition of the coordinates ${{\textbf {X}}}^h$, i.e., a square or rectangle, such that $\Omega _0\subset \Omega _\square$. The remaining part of the extended domain $\Omega _f\subset \Omega _\square$, for which $\Omega _0\cap \Omega _f=\emptyset$, $\Omega _\square =\Omega _0\cup \Omega _f$ holds, is considered as fictitious. However, since the weak form Eq. (13) should only hold in $\Omega _0$, immersed methods are faced with the challenges of

- Distinguishing between $\Omega _0$ and $\Omega _f$ and ensuring that the boundaries of $\Omega _0$ are accurately resolved when computing the integrals in Eq. (25) [70],
- Ensuring that the problem remains well-posed also for the control points that lie outside $\Omega _0$ in the fictitious domain $\Omega _f$, i.e., ensuring that the tangent stiffness matrix in Eq. (30) remains positive definite [14–16], and
- Applying Dirichlet boundary conditions on $\Gamma _\textrm{D}$, since the discretization is not interpolatory on the boundary of the physical domain $\partial \Omega _0$ [23].

### Boundary conformal quadrature

In this work, we assume a spline-based boundary representation (B-rep) of the boundaries of the physical domain as a closed loop of B-spline or NURBS curves, $\partial \Omega _0=\bigcup _c\gamma _c$, see [2], as illustrated in Fig. 2a. Similarly, also a level-set representation could be employed [15].

To achieve an accurate integration over the physical domain $\Omega _0\subset \Omega _\square$, based on this representation of $\partial \Omega _0$, the B-rep is split according to the vertical and horizontal knot lines of the parametric domain, see Fig. 2, in an operation denoted as slicing. This involves computing the intersections among the boundary curves and knot lines, which must be performed with a sufficiently high precision as subsequent operations, including the accuracy of the ulterior PDE analyses, will be controlled by such precision^1^. As a result of the slicing, the extended domain $\Omega _\square$ is decomposed into elements $\Omega _\square =\bigcup _e \Omega ^e$ that are classified into three families, see Fig. 2b:

- Active ($$\Omega ^e\subset \Omega _0$$),
- Trimmed ($$\Omega ^e\cap \Omega _0\ne \emptyset \;\wedge \;\Omega ^e\cap \Omega _f\ne \emptyset $$),
- Non-active ($$\Omega ^e\subset \Omega _f$$).

On active and non-active elements, the discretized weak form is integrated regularly with the standard Gauss quadrature rule, c.f. Eq. (28). For the trimmed elements, that are represented again through B-reps, we adopt the boundary conformal quadrature method [86]. The technique depends on re-parameterization of trimmed elements and consists of three stages, approximation, decomposition, and re-parameterization:

1. In the approximation step, for every trimmed element, the B-rep segment of the boundary of the physical domain is approximated by a Bézier curve with a polynomial degree *p* of the same order as the spline discretization Eq. (23), see Fig. 2b. As proved in [2], to approximate the boundaries with the same degree *p* as the one used to discretize the solution is enough to keep the consistency error under control and guarantee optimality in the case of elliptic problems.
2. In the decomposition step, every trimmed element is decomposed into the active and non-active parts, as indicated by the different color shades in Fig. 2b. If a such an integration cell is not a topological triangle or quad, such as the pentagonal active part of the center element in Fig. 2b, it is further decomposed by dividing the cell along the trimming curve into triangular or quadrilateral sub-cells, see Fig. 2c. Further details are provided in [86].
3. Finally, the integration cells are re-parameterized as B-spline surfaces of degree *p* and the quadrature points are seeded in the cells according to a Gauss rule with $n_{qp}=(p+1)\times (p+1)$ points, see Fig. 2c.

In this way, the boundary $\partial \Omega _0$ and the integration over $\Omega _0$ are resolved numerically exact up to the approximation error that stems from the re-parameterization of the trimmed cell boundaries. Note that the trimmed elements are only decomposed and re-parameterized for the purpose of determining the quadrature point locations, which does not affect the discretization and the number of degrees of freedom. A similar re-parameterization approach was also proposed in [79]. There, the trimming curves and thus the re-parameterized patches are approximated with often high orders, and the trimmed patches are fully reconstructed in a boundary conformal fashion. However, in the immersed approach presented here, the boundary is re-parametrized using the same degree as the one used for the solution discretization, for the sole purpose of generating quadrature points, what guarantees optimal approximation properties [2].

While the use of boundary conformal quadrature points ensures that the integrals involved in the discretized weak form, see Eq. (28), can be accurately calculated, it still must be considered that the tangent stiffness matrix  remains positive definite. As the support of some B-spline basis functions may lie completely outside the physical domain $\Omega _0$, i.e., completely inside the fictitious domain $\Omega _f$, integrating only over $\Omega _0$ would lead to 0-entries in . Thus, the integrals are in fact integrated over $\Omega _\square$, with boundary conformal quadrature points in the physical and in the non-physical parts of the trimmed elements, but the strain energy, and consequently all integrals terms involved in , and , are penalized with a penalty factor $\alpha$:

32 $$\begin{aligned} \Psi ({{\textbf {F}}}) \equiv \alpha \, \Psi ({{\textbf {F}}}), \quad \alpha = {\left\{ \begin{array}{ll} 1, &  {{\textbf {X}}}\in \Omega _0\\ 10^{-q}, &  {{\textbf {X}}}\in \Omega _f \end{array}\right. }. \end{aligned}$$

If the integration point lies in the physical domain $\Omega _0$, then $\alpha =1$, otherwise, if the integration point lies in the non-physical, fictitious domain $\Omega _f$, then $\alpha$ takes a sufficiently small value to have a non-significant contribution in the non-physical domain, where $q \in [{-6}, {-15}]$, as typically used also in other works [75]. It is noteworthy that in small deformation problems, the fictitious domain can be disregarded by fixing the degrees of freedom associated solely with non-active non-trimmed elements. Consequently, no integration points are required for the fictitious domain ($\alpha =0$). However, for nonlinear problems, inclusion of the fictitious domain in the integration process is essential to enhance the condition number of the overall system and ensure stability for large deformations.

### Deformation map resetting

Since the penalization of the fictitious domain according to Eq. (32) models the non-physical part as a very soft hyperelastic material, nonphysically large deformations may occur there in finite strain problems. These extremely large displacements in the non-physical domain contribute to problems for the uniqueness of the deformation map, which also limit the ability to use very small values for the penalization factor $\alpha$. Since this influences the robustness of the method, we adopt the modified formulation based on repeated deformation resetting in the fictitious domain as introduced in [77]. The idea can be explained simply as following: if an integration point lies in the non-physical domain, we reset the current configuration to the reference configuration in each Newton–Raphson step as

33 $$\begin{aligned} \varvec{\varphi }({{\textbf {X}}}) = {\left\{ \begin{array}{ll} {{\textbf {x}}}, &  \text {if } {{\textbf {X}}}\in \Omega _0\\ {{\textbf {X}}}, &  \text {if } {{\textbf {X}}}\in \Omega _f \end{array}\right. }. \end{aligned}$$

This can simply be implemented by setting the deformation gradient to identity for integration points in the non-physical domain as

34 $$\begin{aligned} {{\textbf {F}}}({{\textbf {X}}}) = {\left\{ \begin{array}{ll} {{\textbf {I}}}+\nabla _{{\textbf {X}}}{{\textbf {u}}}, &  \text {if } {{\textbf {X}}}\in \Omega _0\\ {{\textbf {I}}}, &  \text {if } {{\textbf {X}}}\in \Omega _f \end{array}\right. }. \end{aligned}$$

In this way, even though the control points in the fictitious domain may undergo large, potentially unphysical deformations, the resulting strains and stresses are neglected in the computation of the residual and stiffness matrix. This is analogous to always assuming linear elastic behavior in the fictitious domain in every Newton iteration. As a consequence, large deformations in the fictitious domain do not negatively impact the overall displacements and solutions in the physical domain. This method thus allows the use of very small penalization factors $\alpha \le 10^{-10}$, while maintaining a stable formulation.

### Weak imposition of essential boundary conditions

Applying boundary conditions for boundary conformal FEM and IGA is straightforward for both natural (Neumann, traction) boundary conditions and essential (Dirichlet, displacement) boundary conditions (BC), as the physical boundaries coincide with nodes or control points on the boundaries of the mesh. Unfortunately, this is generally not the case when using fictitious domain methods.

The natural BCs on $\Gamma _\textrm{N}\subset \partial \Omega _0$ enter the weak form and thus the external force vector  through surface integrals, see Eq. (25). They can be integrated by providing a local surface (i.e., here curve) mesh representing the boundary surface explicitly. Here, this directly provided by the curves $\gamma _c$, which define the B-rep of the physical domain $\Omega _0$. Furthermore, if it does not coincide with $\partial \Omega _0$, we assume zero-traction BC on the boundaries of the extended domain $\partial \Omega _\square$.

#### Penalty method

If the physical domain boundaries cut the elements, imposing the essential boundary conditions ${{\textbf {u}}}={{\bar{{{\textbf {u}}}}}}$ on $\Gamma _\textrm{D}\subset \partial \Omega _0$, see Eq. (12), strongly is not possible. Hence, a weak enforcement technique is needed. One idea is to modify the weak form without constraining the displacement field. The penalty method [5, 92] is one of the most widely used methods for weakly imposing DBCs. Its core idea is to add an additional energy term to the variational form Eq. (13), which does not directly constrain the displacement field and only penalizes violation of the essential BC:

35 $$\begin{aligned} \delta {\mathcal {W}}^{\text {pen}} ({{\textbf {u}}},\delta {{\textbf {u}}}) = \beta \int _{\Gamma _\textrm{D}} \delta {{\textbf {u}}}\cdot ({{\textbf {u}}}- {{\bar{{{\textbf {u}}}}}}) \ \text {d}A, \end{aligned}$$

where $\beta \gg 0$ is a penalty parameter, which should be selected to be sufficiently large to impose the essential BC.

The discretized virtual work of the penalty term results in additional terms for the internal and external force vectors, see Eq. (26), as

36 $$\begin{aligned} \begin{aligned} {{\textbf {f}}}_{\text {int},i}^{\, \text {pen}}({{\textbf {u}}}^h)&= \beta \ \int _{\Gamma _\textrm{D}} N_i\,{{\textbf {u}}}\ \text {d}A ,\\ {{\textbf {f}}}_{\text {ext},i}^{\, \text {pen}}&= \beta \ \int _{\Gamma _\textrm{D}} N_i \,{{\bar{{{\textbf {u}}}}}} \ \text {d}A. \end{aligned} \end{aligned}$$

Consequently, there is also an additional contribution to the tangent stiffness matrix, see Eq. (30), as

37 $$\begin{aligned} {{\textbf {K}}}_{ij}^{\text {pen}} = \beta \int _{\Gamma _\textrm{D}} N_i N_j\ \text {d}A. \end{aligned}$$

The penalty method does not add any additional unknowns to the problem formulation and preserves the positive definiteness, symmetry, and band structure of the stiffness matrix. However, the penalty parameter $\beta$ must be chosen sufficiently large to enforce the Dirichlet boundary conditions, while also avoiding a significant deterioration in the conditioning of the system.

#### Nitsche’s method

Nitsche’s method can be applied for weakly imposing Dirichlet BC [58]. It has been used in the context of immersed boundary and fictitious domain methods, for mesh-free methods [24], cut elements [10], in the FCM for small deformation [72], and for linear thermoelasticity [90].

In Nitsche’s method, in addition to a penalty term, consistency and symmetry terms are added to the variational form from Eq. (13) as

38 $$\begin{aligned} \begin{aligned} \delta {\mathcal {W}}^{\text {Nit}}({{\textbf {u}}},\delta {{\textbf {u}}})&= \beta \int _{\Gamma _{\text {D}}} \delta {{\textbf {u}}}\cdot ({{\textbf {u}}}-{{\bar{{{\textbf {u}}}}}}) \, \text {d}A-\int _{\Gamma _{\text {D}}} \delta {{\textbf {u}}}\cdot ({{\textbf {P}}}\cdot {{\textbf {n}}}) \, \text {d}A-\int _{\Gamma _{\text {D}}} \delta ({{\textbf {P}}}\cdot {{\textbf {n}}}) \cdot ({{\textbf {u}}}-{{\bar{{{\textbf {u}}}}}}) \, \text {d}A . \end{aligned} \end{aligned}$$

Here, the stabilization parameter $\beta$ is chosen based on the grid discretization, polynomial order, and material parameters.

Compared to the penalty method, Nitsche’s method preserves the weak form consistency. In addition, it shares with the penalty method the advantage of preserving the symmetry.

However, in the case of imposing Dirichlet boundary conditions on badly cut elements (those where a large part of the element is inactive), stability cannot be guaranteed a priori. In such cases, stabilization techniques may be needed, such as the minimal stabilization strategy for proposed in [9]. Here, we rely on the fact that the penalization in the fictitious domain, c.f. Eq. (32), improves both the stability and the conditioning of the system [16], as no numerical instabilities were observed in the numerical experiments presented in Sect. 4.

Since Eq. (38) includes the term $\delta ({{\textbf {P}}}\cdot {{\textbf {n}}})$, its linearization requires the second derivatives of ${{\textbf {P}}}$ w.r.t. ${{\textbf {F}}}$, i.e., $\partial ^3\Psi /\partial {{\textbf {F}}}^3$. These expressions are generally difficult to derive and compute; and thus, we only apply Nitsche’s method for linear elasticity, where it can be formulated as

39 $$\begin{aligned} \begin{aligned} \delta {\mathcal {W}}^{\text {lin,Nit}}({{\textbf {u}}},\delta {{\textbf {u}}})&= \beta \int _{\Gamma _{\text {D}}} \delta {{\textbf {u}}}\cdot ({{\textbf {u}}}-{{\bar{{{\textbf {u}}}}}}) \, \text {d}A-\int _{\Gamma _{\text {D}}} \delta {{\textbf {u}}}\cdot \big (\varvec{\varepsilon }({{\textbf {u}}}):{\mathbb {C}}\cdot {{\textbf {n}}}\big ) \, \text {d}A-\int _{\Gamma _{\text {D}}} \big (\varvec{\varepsilon }(\delta {{\textbf {u}}}):{\mathbb {C}}\cdot {{\textbf {n}}}\big ) \cdot ({{\textbf {u}}}-{{\bar{{{\textbf {u}}}}}}) \, \text {d}A . \end{aligned} \end{aligned}$$

### Preconditioning and stability

The application of immersed FEM/IGA methods is often accompanied by stabilization issues due to the weak imposition of boundary conditions. Identifying these stability issues can be challenging. For instance, in [9], unstable scenarios such as high aspect ratio rectangular trimmed elements were examined, and instabilities in the normal gradient term were observed when applying Dirichlet boundary conditions using Nitsche’s method. The authors suggested two stabilization techniques: one based on polynomial extrapolation in the parametric domain and another employing a projection-based stabilization directly in the physical domain. For further reading on this topic, we refer the reader to [22, 33, 39, 54, 55]. However, none of these stability techniques were employed in the current work, as the fictitious domain method implemented here was found to be a sufficient remedy for ensuring stability.

In addition to stability issues, the stiffness matrix can suffer from ill-conditioning due to small cuts, as some basis functions lose their support. Detailed discussions on this can be found in the literature [14, 15, 45, 49]. In the current work, in addition to the fictitious domain method, we implemented a diagonal scaling preconditioner to address this issue. For a linear system ${{\textbf {A}}}{{\textbf {x}}}= {{\textbf {b}}}$, where ${{\textbf {A}}}\in {\mathbb {R}}^{n \times n}$ is the system stiffness matrix and ${{\textbf {x}}}, {{\textbf {b}}}\in {\mathbb {R}}^n$ are the unknown vector and the right-hand side vector, respectively, and $n$ is the system size, we define a diagonal scaling preconditioner ${{\textbf {D}}}$ as

40 $$\begin{aligned} {{\textbf {D}}}= \textrm{diag} \left( 1/\sqrt{A_{11}}, 1/\sqrt{A_{22}}, \ldots , 1/\sqrt{A_{nn}} \right) , \end{aligned}$$

and we rewrite our linear system of equations as

41 $$\begin{aligned} {{\textbf {D}}}{{\textbf {A}}}{{\textbf {D}}}{{\textbf {z}}}= {{\textbf {D}}}{{\textbf {b}}}. \end{aligned}$$

After solving the system for ${{\textbf {z}}}$, the desired unknown vector is calculated as

42 $$\begin{aligned} {{\textbf {x}}}= {{\textbf {D}}}^{-1} {{\textbf {z}}}. \end{aligned}$$

This diagonal preconditioning is observed to be effective for B-splines bases, in contrast to Lagrange bases [14]. Here, the linear system from Eq. (42) is solved using MATLAB’s sparse direct solver, where no issues with conditioning are observed as ${{\textbf {D}}}$ remains positive semi-definite.

## Numerical results

In the following, we apply immersed isogeometric analysis with boundary conformal quadrature (IBC) to 2D plane strain benchmark problems in linear and finite deformation elasticity to demonstrate its efficiency and accuracy. The approach is implemented within the framework of the MATLAB code NLIGA (https://sourceforge.net/projects/nliga/, [18]) and the in-house library based on irit [21] and Open CASCADE [60] for obtaining the boundary conformal quadrature points.

### Plate with circular hole-linear elasticity

To assess the accuracy and efficiency of our proposed approach, we undertake the analysis of a plate with a circular hole subjected to in-plane tension [38].

**Fig. 3:**
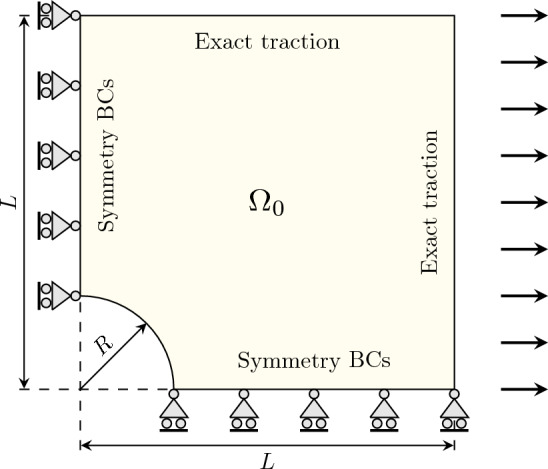
Schematic drawing for the plate with hole problem under in-plane load

Due to the inherent double symmetry of the problem, we focus on solving a quarter of the structure, as illustrated in Fig. 3. The plate, composed of an isotropic linear elastic material with a Young’s modulus of $E=10^5$ and a Poisson’s ratio of $\nu =0.3$, incorporates a hole with a radius $R=1$, centered at the origin, and a plate length $2L=8$ (i.e., half the plate length is $L=4$, as depicted in Fig. 3). Symmetry BC are applied on the left side of the quarter plate (${\bar{u}}_1=0$, $t_2=0$) and on the bottom side (${\bar{u}}_2=0$, $t_1=0$). A traction load is applied to the right and top sides of the quarter plate, which corresponds to the analytical solution for the Cauchy stresses as ${{\textbf {t}}}=\varvec{\sigma }\cdot {{\textbf {n}}}$. Given the far-field traction $T_x$, the analytical solution can be formulated in cylindrical coordinates $(r,\theta )$, see [73], as

43 $$\begin{aligned} \begin{aligned} u_{x}=&\frac{RT_x}{8\mu } \bigg ( \frac{r}{R} (\kappa +1)\cos {\theta } - \frac{2R^3}{r^3} \cos {3\theta }+\frac{2R}{r} \big ((\kappa +1)\cos {\theta }+\cos {3\theta }\big ) \bigg ), \\ u_{y}=&\frac{RT_x}{8\mu } \bigg ( \frac{r}{R} (\kappa -3)\sin {\theta } - \frac{2R^3}{r^3} \sin {3\theta }+\frac{2R}{r} \big ((1-\kappa )\sin {\theta }+\sin {3\theta }\big ) \bigg ), \end{aligned} \end{aligned}$$

where $\mu$ and $\kappa$ are the shear modulus and a secondary elasticity constant, respectively, and both defined for plane stress problems as:

44 $$\begin{aligned} \mu =\frac{E}{2(1+\nu )} , \qquad \kappa =\frac{3-\nu }{1+\nu }. \end{aligned}$$

The resulting Cauchy stresses can be expressed as:

45 $$\begin{aligned} \begin{aligned}&\sigma _{rr}=\frac{T_x}{2}\left( 1-\frac{R^2}{r^2}\right) +\frac{T_x}{2}\left( 1-\frac{4R^2}{r^2}+\frac{3R^4}{r^4}\right) \cos {2\theta }, \\&\sigma _{\theta \theta }=\frac{T_x}{2}\left( 1+\frac{R^2}{r^2} \right) -\frac{T_x}{2}\left( 1+\frac{3R^4}{r^4} \right) \cos {2\theta }, \\&\tau _{r\theta }=-\frac{T_x}{2}\left( 1+\frac{2R^2}{r^2}-\frac{3R^4}{r^4} \right) \sin {2\theta }. \end{aligned} \end{aligned}$$

To asses the errors of the numerical solutions in the following studies, we employ the $L_2$-norm of the displacement error, which is computed as

46 $$\begin{aligned} {\Vert {{\textbf {u}}}-{{\textbf {u}}}^{h} \Vert }_{L_2}^2= \int _{\Omega _0} {\Vert {{\textbf {u}}}-{{\textbf {u}}}^{h} \Vert }_{2}^2 \ \ \text {d}V . \end{aligned}$$

First, we immerse the physical domain $\Omega _0$ of the quarter plate with hole into the square domain $\Omega _\square =[0,4]^2$. In this case, the physical domain boundaries $\partial \Omega _0$ coincide with the boundaries of the extended domain $\partial \Omega _\square$, except at the circular hole, the only immersed part. Thus, it is possible to strongly impose the Dirichlet boundary conditions by eliminating them. Furthermore, for the integration over the fictitious domain, we use the penalization parameter $\alpha =10^{-10}$. The number of Gauss points in the integration cells is set to $p+1$ in each direction.

**Fig. 4:**
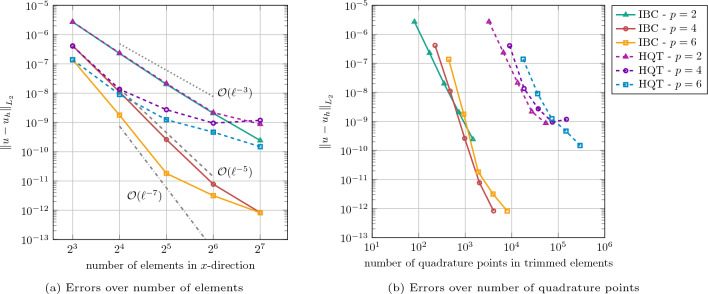
Comparison of convergence behaviors of immersed IGA with IBC and HQT quadrature for the linear elastic plate with hole problem in the $L^2$-norm of the displacement error

**Fig. 5:**
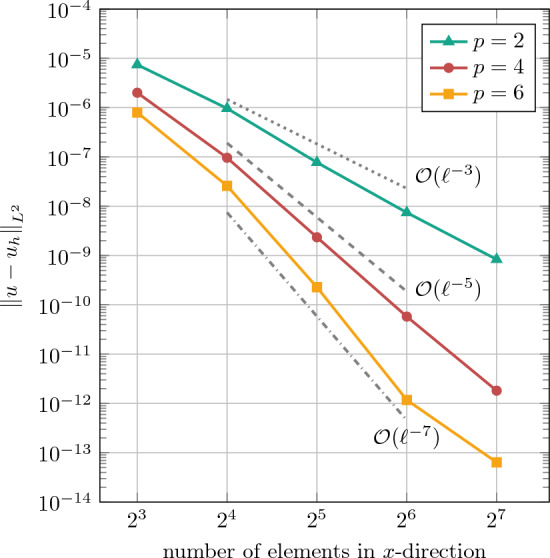
Convergence behaviors of fully immersed IGA with IBC and weak BC for the linear elastic plate with hole problem in the $L^2$-norm of the displacement error

**Fig. 6:**
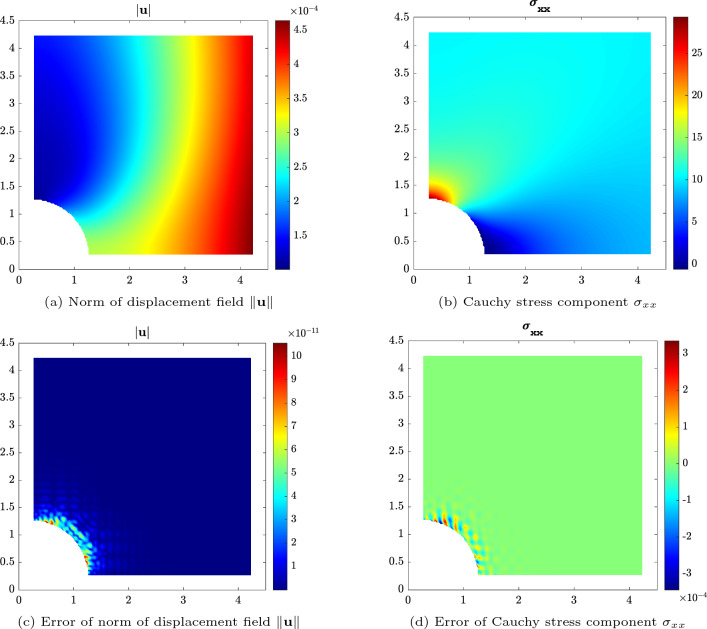
Displacement and stress plots for the fully immersed IBC solution of the linear elastic plate with hole problem. The background mesh is constructed by $32\times 32$ elements and with polynomial order $p=6$

The *h*-refinement analysis at different polynomial degrees in Fig. 4a shows proper rates of convergence of order $p+1$ for the IBC with basis functions of order $p=\left\{ 2,4,6\right\}$. The $L_2$-norm of the displacement error approaches the machine error in the case of $p=6$ and a number of elements in each direction of 128.

Figure 4a also shows the results for solving the same problem using isogeometric FCM with hierarchical quadtree refinement for integration (abbreviated as “HQT”), where a tree depth of 5 and $p+1$ Gauss points per cell are used. For the case of basis function with a degree $p=2$, both the proposed IBC and HQT show the same errors and rate of convergence with a slight deviation at large numbers of elements. However, for basis functions with degrees $p=\{4,6\}$, the current work consistently outperforms HQT in terms of convergence rates. For higher number of elements at higher degrees, HQT errors plateau as the overall error is then dominated by the quadrature error and even more quadtree levels would be required to increase accuracy. This is further illustrated in Fig. 4b, where the same $L_2$-errors of both IBC and HQT are plotted over the number of Gauss points used in the trimmed elements. It can be seen that the number of quadrature points required using the IBC approach—and thus the overall computational effort of the assembly—is orders of magnitude smaller than in the HQT case.

.

**Fig. 7:**
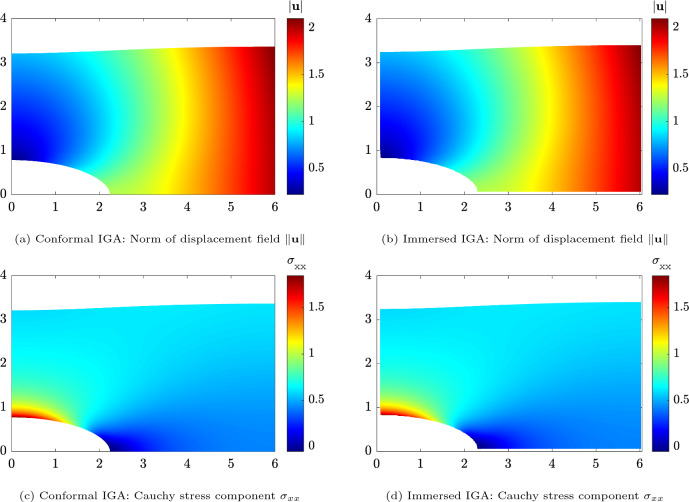
Displacements and stresses of the hyperelastic plate with hole obtained by conformal IGA (left column) and immersed IGA with boundary conformal quadrature (right column), plotted over the deformed configuration. Degree $p=6$ was considered in both cases, while $128\times 64$ elements were used in the conformal case and $32\times 32$ background elements for the immersed one

Second, the physical domain $\Omega _0$ is fully immersed into the square domain $\Omega _\square =[0,4.5]^2$ with a gap of 0.25 around the plate of side length $L=4$.

Thus, the problem must be solved by weakly imposing Dirichlet BCs, which we do here using Nitsche’s method, see Sect. 3.7.2. The stabilization parameter is set to $\beta =6Ep^2/h$, where *h* is the element length, see [3]. In contrast to the previous case, where the stiffness matrix calculations include the fictitious domain, we now exclude all elements belonging to this domain from the assembly loop as well as the inactive part of the trimmed elements, i.e., we restrict the domain of integration to $\Omega _0$. Corresponding degrees of freedom are fixed. By this, we effectively reduce computational time and eliminate the need for a penalization factor $\alpha$ or a threshold to mitigate the fictitious domain’s impact.

For this fully immersed case with weak BC enforcement, a *h*-refinement analysis at different polynomial degrees is carried out in Fig. 5. We observe proper rates of convergence for the $L_2$-norm of the displacement error, which confirms the correct implementation and the efficiency of the IBC method. Comparing these results with the previous case in Fig. 4a, the errors are slightly smaller as the fictitious domain effect is eliminated without using a penalization factor.

As an exemplary result, for a discretization with $p=6$ and $32\times 32$ elements, the norm of the displacement field and the contour of Cauchy stress component $\sigma _{xx}$ are displayed over the reference configuration in Fig. 6a and b, respectively. The plots showcase the anticipated displacement and stress fields, which is further illustrated by Fig. 6c and d, where the corresponding errors between the proposed method and the exact solution for the displacement field and stress are shown. Overall, the error levels are very low and, as expected, the maximum errors are concentrated in and near the trimmed elements around the hole.

### Plate with circular hole-finite deformation elasticity

Now, we verify and investigate the immersed IGA with boundary conformal quadrature in the finite deformation regime with hyperelastic material behavior.

As a benchmark problem, we consider again the plate with hole example from Sect. 4.1, see Fig. 3, but with slightly different boundary conditions: on the right side, *x*-direction displacements are prescribed as ${\bar{u}}_1=2$ while $t_2=0$, and on the top side Neumann BC are used with ${{\textbf {t}}}={\textbf{0}}$. Furthermore, the hyperelastic material is modeled using a Mooney–Rivlin material, see Eq. (10), characterized by the parameters $A_{10}=0.1863$ and $A_{01}=0.00979$.

The extended domain is chosen as $\Omega _\square =[0,4.1]^2$, with a gap of 0.05 around the plate of side length $L=4$. To mitigate the influence of the fictitious domain, a penalization factor of $\alpha =10^{-15}$ is employed. For the weak imposition of BCs, we utilize the penalty method with a sufficiently large penalty parameter $\beta ^{\text {pen}}=C\,6p^{2}/h$ to rigorously enforce the boundary conditions, where $C=2.965856\times 10^4$ is selected experimentally such that it does not affect the solver stability. The nonlinear problem is solved in 3 load steps using a Newton–Raphson method with a tolerance of $10^{-15}$ set for each iteration.

To facilitate a thorough comparison of results, we computed a reference, overkill solution using a boundary conformal NURBS discretization with $p=6$ and $8192=128\times 64$ elements, which is already converged close to machine precision. The norm of the displacement field and the Cauchy stress component $\sigma _{xx}$ over the deformed configuration obtained using conformal IGA and the proposed IBC with $p=6$ and $32\times 32$ background elements are depicted in Fig. 7. Notably, both solutions reveal a remarkable level of agreement and are visually indistinguishable.

**Fig. 8:**
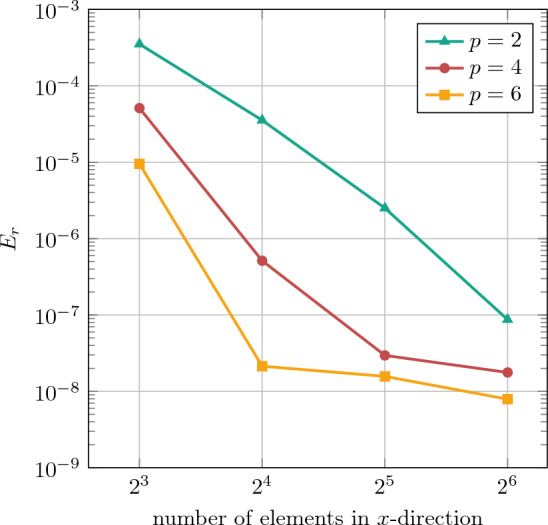
Convergence behaviors of immersed IGA with IBC for the hyperelastic plate with hole problem in the relative strain energy error $E_r$ for *h*-refinement with $p=\{2,4,6\}$

Furthermore, the accuracy of the numerical solution using conformal IGA and IBC is measured by the total strain energy

47 $$\begin{aligned} {\mathcal {W}}_{\text {int}}({{\textbf {u}}}^h)=\int _{\Omega _0} \Psi ({\varvec{F}})\,\, \text {d}V, \end{aligned}$$

and the relative error is evaluated as

48 $$\begin{aligned} E_r = \left| \frac{{\mathcal {W}}_{\text {int}}^{\text {IGA}}-{\mathcal {W}}_{\text {int}}^{\text {IBC}}}{{\mathcal {W}}_{\text {int}}^{\text {IGA}}}\right| , \end{aligned}$$

where ${\mathcal {W}}_{\text {int}}^{\text {IGA}}=1.59859278592585$. A convergence study is performed by evaluating the relative error $E_r$ for employing both *h*- and *k*-refinements, as illustrated in Fig. 8. For the three polynomial orders considered, convergence is observed, reaching values around $10^{-8}$. Also here, (initially) the desired convergence behaviors are obtained using the IBC approach. It is noteworthy that the error plateaus for higher refinements, which can potentially be attributed to using the penalty method for weakly enforcing the Dirichlet BC and the choice of penalty parameter $\beta ^{\text {pen}}$.

**Fig. 9:**
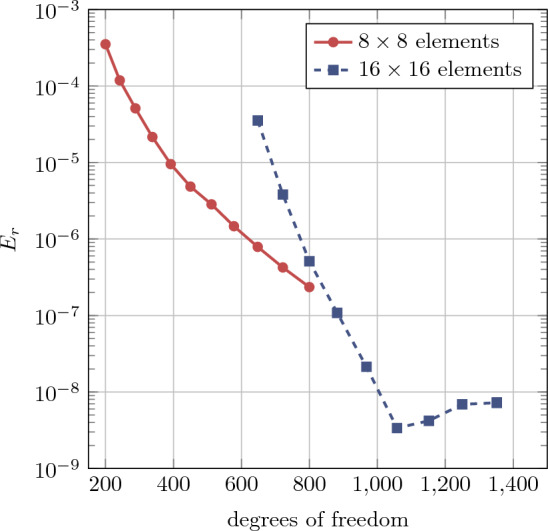
Convergence behavior for the hyperelastic plate with hole in *k*-refinement for uniformly increasing $p=\{2,3,\ldots ,12\}$ with a background mesh of $8 \times 8$ elements and for $p=\{2,3,\ldots ,10\}$ with $16\times 16$ elements

Further, a uniform *p*-refinement (or rather *k*-refinement, where the maximal element continuity of $\text {C}^{p-1}$ is preserved) analysis is conducted with background meshes of $8\times 8$ and $16 \times 16$ elements while the polynomial degree is uniformly increased in $p=\{2,3,\ldots ,12\}$ and $p=\{2,3,\ldots ,10\}$, respectively. The results shown in Fig. 9 exhibit exponential convergence for the relative energy error $E_r$. Similar convergence behavior is found in [75] using the FCM for linear elasticity.

**Fig. 10:**
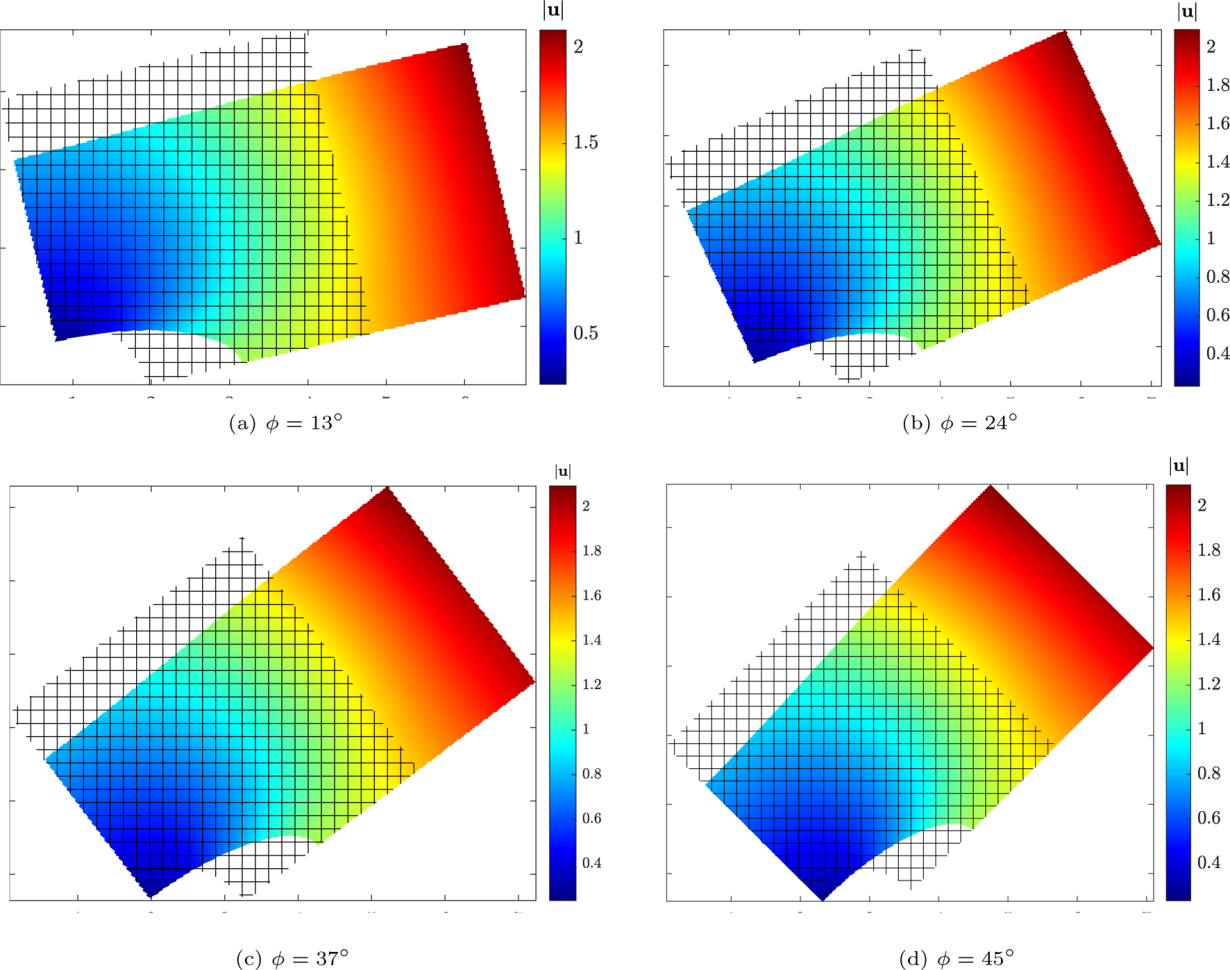
Displacement distributions of hyperelastic plate with a hole, obtained using immersed IGA with boundary conformal quadrature, displayed over the deformed configuration at various rotation angles of the physical domain. Trimmed initial meshes are outlined in black. The analysis was conducted with a polynomial degree of $p=4$ and a background mesh consisting of $32\times 32$ elements

**Fig. 11:**
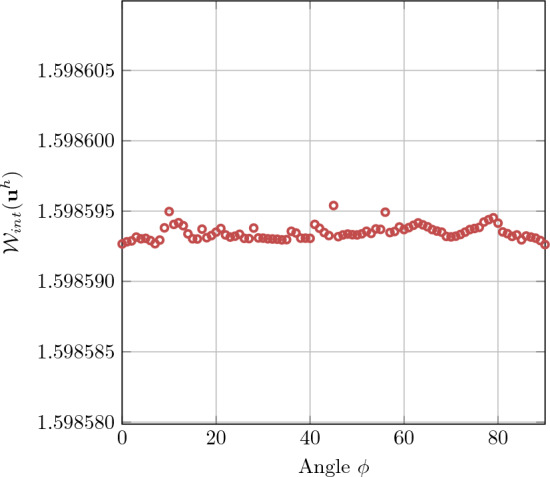
Comparison of the internal energy for the hyperelastic plate with hole problem at different rotation angles of the immersed physical domain

To further explore the robustness of the proposed immersed IGA with boundary conformal quadrature, we rotate the physical domain $\Omega _0$ relative to the extended domain $\Omega _\square$ by an angle $\phi$, which leads to arbitrarily cut elements and kinks. The dimensions of the extended domain in both directions are set to the diagonal of the physical domain plus an offset value $(\sqrt{2}L + 2 \times \text {offset})$, ensuring complete immersion of the physical domain. The Dirichlet boundary conditions are applied weakly using the penalty method. Due to the boundaries no longer aligning with the Cartesian grid, special treatment is necessary to account for the rotation. We implement modifications to the penalty method as outlined in [52], maintaining the penalty parameter consistent with the previous unrotated case. The physical domain is rotated by angles $\phi = \{0, 1, 2, 3, \ldots , 90\} \cdot \pi /180$. The deformed configuration is obtained using immersed IGA with a polynomial degree of $p = 4$ and a $32 \times 32$ background mesh, as depicted in Fig. 10. For each rotation angle, the energy norm is calculated and presented in Fig. 11. In each case, some elements are severely cut, with only a small portion lying within the physical domain. The results indicate that the energy norm remains approximately constant across all rotation angles, demonstrating that the present method provides accurate integration points and robust numerical results even in the presence of elements with minimal contribution to the physical domain.

### Ring plate with hole

**Fig. 12:**
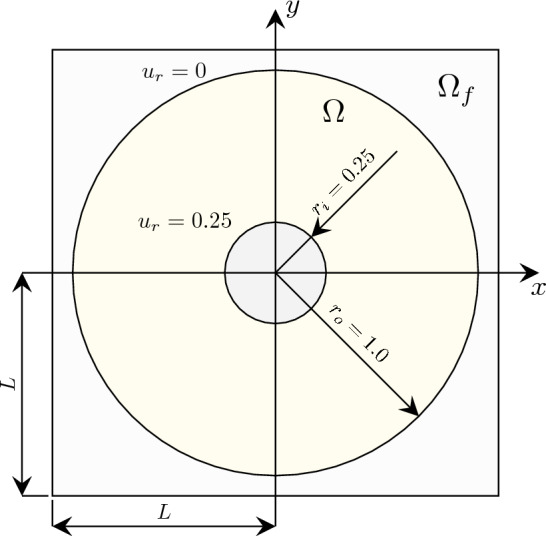
Schematic drawing for the ring plate with hole under internal radial pressure

To rigorously evaluate the imposition of Dirichlet boundary conditions, the problem of a ring plate with a hole is analyzed, in which both homogeneous and non-homogeneous conditions are applied to curved boundaries. As in [77], the outer radius of the plate is fixed, while a radial displacement of $u_r = 0.25$ is imposed on the inner radius. The extended domain length is set to $L = 2.05$, with inner and outer radii $u_i = 0.25$ and $u_o = 1$, respectively, as depicted in Fig. 12. Here, the material model and properties as specified in Fig. 4.2 are employed.

**Fig. 13:**
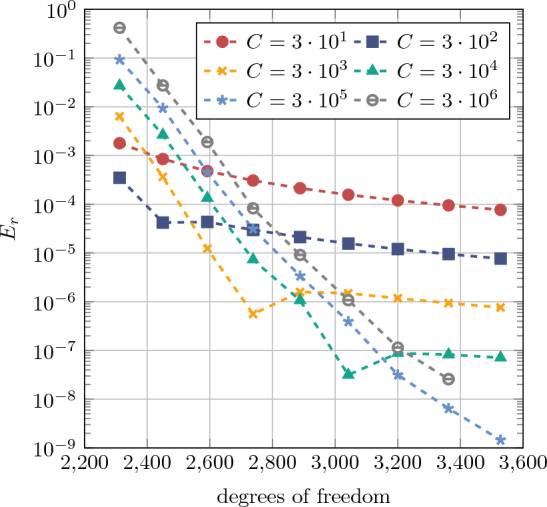
Convergence behavior for the hyperelastic ring plate with hole in *k*-refinement for uniformly increasing $p=\{2,3,\ldots ,10\}$ with a background mesh of $32 \times 32$ elements at different penalty constants *C*

The Dirichlet boundary conditions are weakly enforced using the penalty method, with the penalty parameter defined as $\beta ^{\text {pen}}=C\,6p^{2}/h$ as in Sect. 4.2. The penalty constant *C* is varied as $C=3\cdot 10^q$ with $q=\{1,\ldots ,6\}$ to investigate its effect on the accuracy and convergence rate. The Newton solver configuration is maintained as in the previous section. The results from the IBC method are compared with those from a conformal IGA solution using 16,384 elements of polynomial degree $p = 3$, yielding a total strain energy of ${\mathcal {W}}_{\text {int}}^{\text {IGA}} = 0.26870160930784$.

A *k*-refinement analysis was conducted using a background mesh of $32 \times 32$ elements, with the polynomial degree uniformly increased $p=\{2, 3, \ldots , 10\}$. The results, presented in Fig. 13, are based on the relative error in total strain energy $E_r$, as defined in Eq. (47).

As in previous section, it can be observed that the accuracy of the solution tends to plateau at higher polynomial degrees. Nevertheless, an exponential rate of convergence is evident for sufficiently large values of the penalty parameter *C*. Notably, for the case with $C=3 \cdot 10^6$, the error increased compared to the case with $C=3 \cdot 10^5$. Furthermore, for the case with $C=3 \cdot 10^6$, no converged solution was obtained for polynomial degrees higher than $p=9$. It is obvious that the accuracy depends mainly on the penalty parameter value. Furthermore, numerical experiment revealed no dependence on the number of integration points needed. However, more detailed investigation for the optimal penalty parameter value is needed.

### Homogenization of a microstructure

In this subsection, we want to demonstrate the application of the proposed IBC method to the numerical homogenization of microstructured materials subject to finite deformations.

The numerical simulation of microstructured materials becomes computationally infeasible when considering the full resolution of the micro-scale, in particular for nonlinear problems. Thus, multiscale simulation using homogenization is a crucial technique, which involves evaluating the effective response of a representative volume element (RVE). The micro-to-macro transition must adhere to the Hill-Mandel condition [32], which necessitates that the average variation work of the RVE equals the variation of the work on the macro-scale [80]. Expressed in a boundary integral form for the first Piola–Kirchhoff stress and the deformation gradient tensor, the Hill-Mandel condition is represented as

49 $$\begin{aligned} \int _{\partial \Omega } ({{\textbf {x}}}-{{\textbf {F}}}^*\cdot {{\textbf {X}}})\cdot ({{\textbf {t}}}- {{\textbf {P}}}^*\cdot {{\textbf {n}}}) \, \text {d}A = 0. \end{aligned}$$

Here, ${{\textbf {F}}}^*$ and ${{\textbf {P}}}^*$ are the effective deformation gradient and first Piola–Kirchhoff stress tensor, respectively, which can be computed as averages over the RVE domain $\Omega _0$ as

50 $$\begin{aligned} \begin{aligned} {{\textbf {F}}}^*&=\langle {{\textbf {F}}}\rangle = \frac{1}{|\Omega _0|} \int _{\Omega _0} {{\textbf {F}}}({{\textbf {X}}}) \ \text {d}V, \\ {{\textbf {P}}}^*&=\langle {{\textbf {P}}}\rangle = \frac{1}{|\Omega _0|} \int _{\Omega _0} {{\textbf {P}}}({{\textbf {X}}}) \ \text {d}V \end{aligned} \end{aligned}$$

**Fig. 14:**
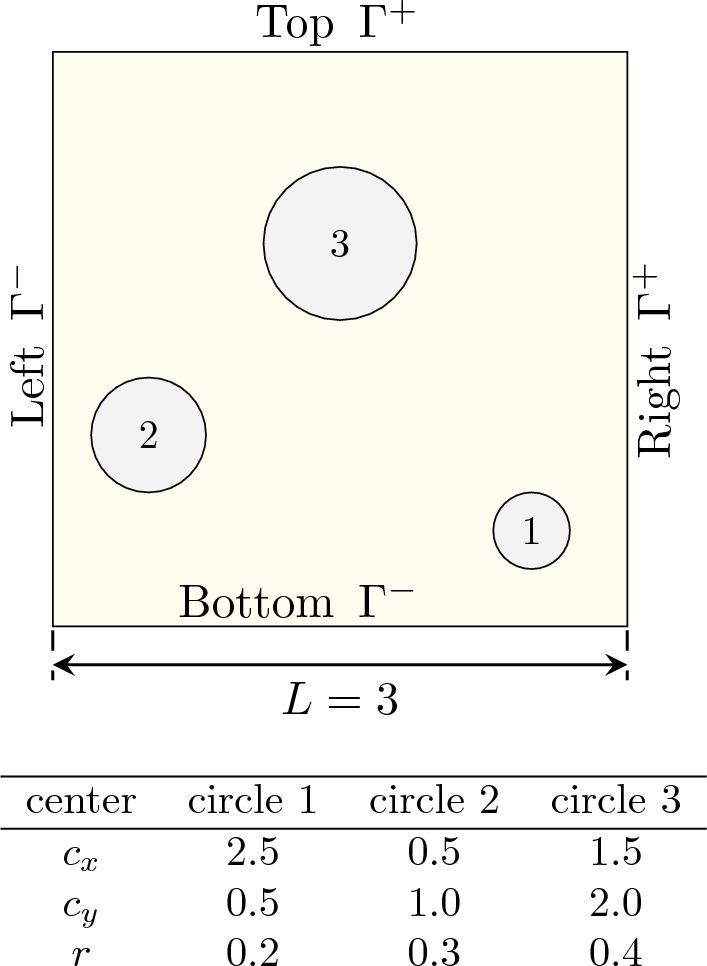
Schematic drawing and geometric parameters for the square RVE with 3 pores

The Hill-Mandel condition can be ensured by using the effective deformation gradient ${{\textbf {F}}}^*$ to define periodic boundary conditions for the RVE problem as

51 $$\begin{aligned} {{\textbf {x}}}^+ - {{\textbf {x}}}^- = {{\textbf {F}}}^*\cdot ({{\textbf {X}}}^+ - {{\textbf {X}}}^-) \end{aligned}$$

for ${{\textbf {X}}}^+\in \Gamma ^+$, ${{\textbf {X}}}^-\in \Gamma ^-$, where $\Gamma ^+$ and $\Gamma ^-$ are complementary subset of the RVE boundary $\partial \Omega _0$, see also Fig. 14.

To demonstrate the straightforward applicability of the IBC-IGA method to nonlinear homogenization, a square RVE with three pores is investigated to serve as an example for a periodic microstructure, see Fig. 14. The pores in the RVE $\Omega _0$ are represented by three circles, which are parameterized as NURBS curves. The matrix material is given by the same hyperelastic material model used in the nonlinear simulations presented in Sect. 4.2. In the case of material inclusions, instead of pores, the circles would be also an active part of the domain. In such a case, the immersed boundary conformal strategy [86] would be a suitable alternative to capture the discontinuous behavior across the material interfaces, see, e.g., [46].

Since the RVE is exactly immersed into the domain $\Omega _\square =[0,L]^2$, the outer boundaries coincide and the periodic boundary conditions are imposed strongly using the elimination method. The numerical homogenization is carried out for two prescribed deformation gradients with uni-directional tension in *x*-direction by ${{\textbf {F}}}^*_\textrm{tens}$ and simple shear by ${{\textbf {F}}}^*_\textrm{shear}$ as

52 $$\begin{aligned} {{\textbf {F}}}^*_\textrm{tens}= \begin{bmatrix} 1+\lambda &  0 \\ 0 &  1 \end{bmatrix}, \qquad {{\textbf {F}}}^*_\textrm{shear}= \begin{bmatrix} 1 &  \lambda \\ 0 &  1 \end{bmatrix}, \end{aligned}$$

where $\lambda \in [0,0.5]$ is the loading parameter.

**Fig. 15:**
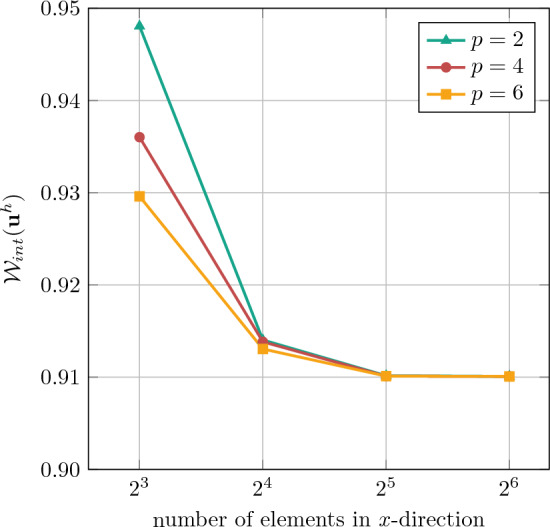
Convergence study for the homogenization of the square RVE with 3 pores in uni-directional tension by ${{\textbf {F}}}^*_\textrm{tens}$ with loading parameter $\lambda =0.5$

First, we investigate the convergence of the IBC method using the same error measure as in the nonlinear plate with hole problem, see Eq. (47). For the uni-directional tension with ${{\textbf {F}}}^*_\textrm{tens}$ with loading parameter $\lambda =0.5$, a convergence study based on *h*-refinement analysis at different polynomial degrees is conducted, as depicted in Fig. 15. The results demonstrate proper convergence for the selected error measure. For all degrees $p\in \{2,4,6\}$, sufficient accuracy is obtained already with 32 to 64 elements per direction.

**Fig. 16:**
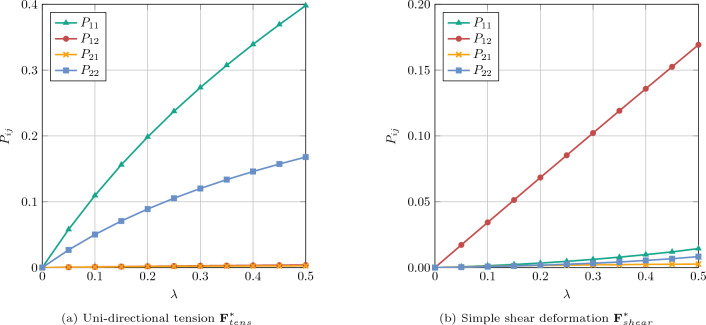
Homogenized first Piola–Kirchhoff stress ${{\textbf {P}}}^*$ for square RVE with pores

**Fig. 17:**
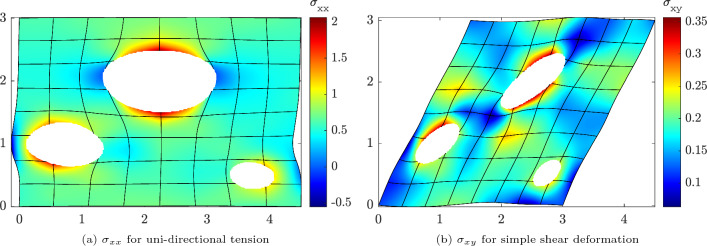
Deformed configurations of homogenized RVE with pores, colored by Cauchy stress

For the subsequent results of the homogenization process with periodic BC, we selected the discretization with $64\times 64$ elements and a polynomial degree of $p=2$. The homogenization of the effective first Piola–Kirchhoff stress ${{\textbf {P}}}^*$ is performed for both uni-directional tension and pure shear deformation cases according to Eq. (52) with $\lambda \in [0, 0.5]$. Figure 16 shows the resulting components of the averaged first Piola–Kirchhoff stress tensor as a function of the loading parameter $\lambda$ for both deformation cases. In the uni-directional tension test, the microstructure exhibits decreasing stiffness with increasing loading, while the shear stress is approximately zero. Conversely, in the shear test, an approximately linear relation between $P_{12}$ and $\lambda$ is evident. Figure 17 shows the corresponding deformed configurations for $\lambda =0.5$, colored by the Cauchy stress components $\sigma _{xx}$ and $\sigma _{xy}$, respectively. Due to the high loading parameter, the circular pores deformed into ellipses. Although this can be considered as a fairly large deformation, the simulation was stable and the solution converged within less than four iterations per Newton–Raphson step using the step size $\Delta \lambda =0.05$. Furthermore, a high level of inhomogeneity of the stress distributions can be observed, but nevertheless stress maxima and minima around the pores can be well-captured by the immersed IGA approach.

### Plate with irregular inclusions

**Fig. 18:**
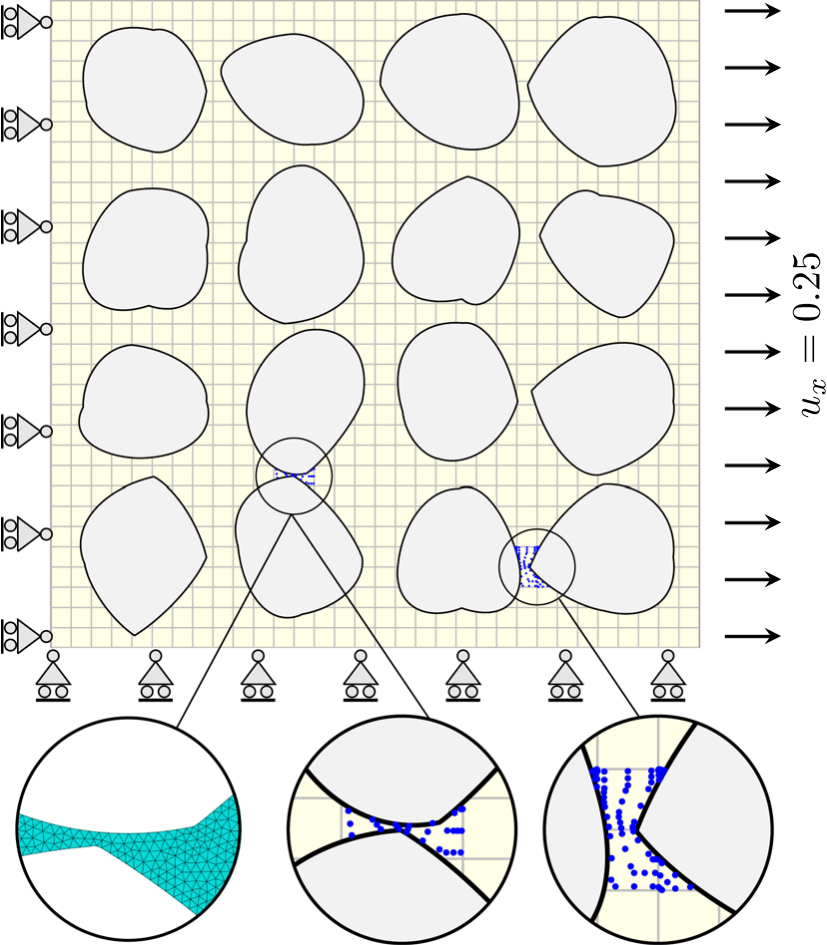
Schematic illustration of the plate with irregular inclusions subjected to tensile loading. Magnified areas highlight examples of geometric complexity and closely spaced inclusions. Left magnified area: triangle mesh from Abaqus. Middle and right magnified areas: the blue dots show the integration points for a rule with $3\times 3$ points per re-parameterized element

To further evaluate the proposed IBC method, we assess its performance on a more complex geometry. We consider a square plate of dimensions $1 \times 1$, containing 16 irregularly shaped inclusions distributed throughout the domain. The plate is subjected to uniaxial tension with a prescribed displacement of $u_x = 0.25$. The length of the extended domain is set equal to that of the physical domain $\Omega _\square =\Omega _0=[0,1]^2$, allowing for the strong imposition of DBCs via elimination. The material model and properties are consistent with those used in the previous nonlinear examples (see Sect. 4.2). The physical domain and boundary conditions are illustrated in Fig. 18.

As shown in the magnifiers in Fig. 18, this problem exhibits several challenging features. The geometry contains inclusions positioned very close to each other, resulting in narrow gaps between them. This leads to elements being trimmed by multiple curves or surfaces. Additionally, the geometry includes multiple kinks and elements where only a small portion of the physical domain remains after trimming.

**Fig. 19:**
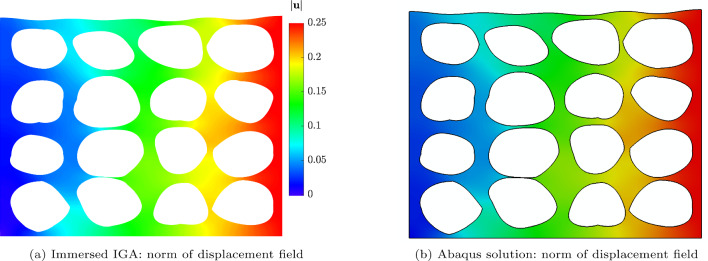
Comparison for the magnitude of displacement for the plate with irregular inclusions. The immersed IGA solution was conducted with background mesh of $32\times 32$ elements and polynomial degree of $p=2$

The results obtained using the IBC method are compared with a reference “overkill” solution computed in Abaqus, where the domain is discretized using 265,085 s-order triangular elements. The strain energy computed in Abaqus is ${\mathcal {W}}_{\text {int}}^{\text {Abaqus}} = 0.00792123$. The resulting deformed geometry is shown in Fig. 19, comparing the displacements obtained from the IBC (here with $32\times 32$ elements an $p=2$) with the Abaqus reference. The overall deformation and the local magnitudes of the displacement field reveal excellent agreement between the immersed IGA and Abaqus solutions. This consistency is particularly evident in the deformation patterns of the inclusions. .

**Fig. 20:**
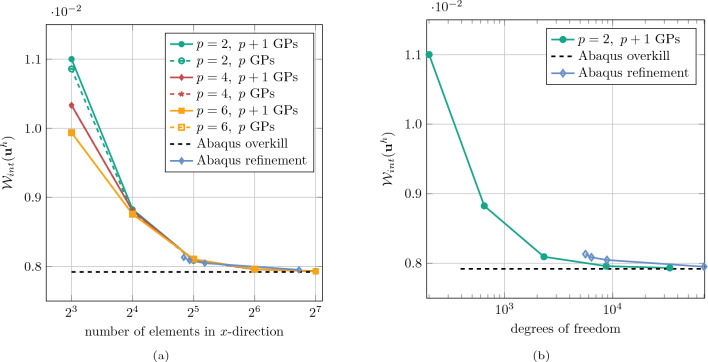
Convergence study for the plate with irregular inclusions. Strain energy against, **a** number of elements in *x*-direction, **b** number of degrees of freedom

Furthermore, an $h$-refinement study was conducted using basis functions of order $p = \{2, 4, 6\}$. For the case with $p = 6$ and a $128 \times 128$ background mesh, the stiffness matrix became nearly singular. This issue was resolved by employing a diagonal preconditioner, see Sect. 3.8. The results, shown in Fig. 20 demonstrate the convergence of the strain energy computed with the IBC method toward the Abaqus reference solution when using $p+1$ integration points per element. The $h$-refinement analysis was repeated using only $p$ integration points per element. The results show nearly the same convergence behavior, confirming the robustness of the IBC method with respect to the choice of integration points.

As demonstrated in Figs. 19 and 20, the proposed immersed IGA method delivers a robust and accurate solution. While *h*-convergence can clearly be observed, due to the very fine geometric features that are not exactly resolved by the immersed discretization, the *p*-convergence behavior is pre-asymptotic and thus the convergence rates for $p>2$ are not optimal. Notably, the IBC-IGA achieved similar accuracy for similar mesh size, but with significantly less geometric modeling effort compared to Abaqus, where extensive manual work is typically required to conform the mesh to complex geometries. In addition to that, Fig. 20b shows that, for an equivalent number of degrees of freedom, the IBC method yields a more accurate solution compared to Abaqus solution, underscoring its efficiency and accuracy.

**Fig. 21:**
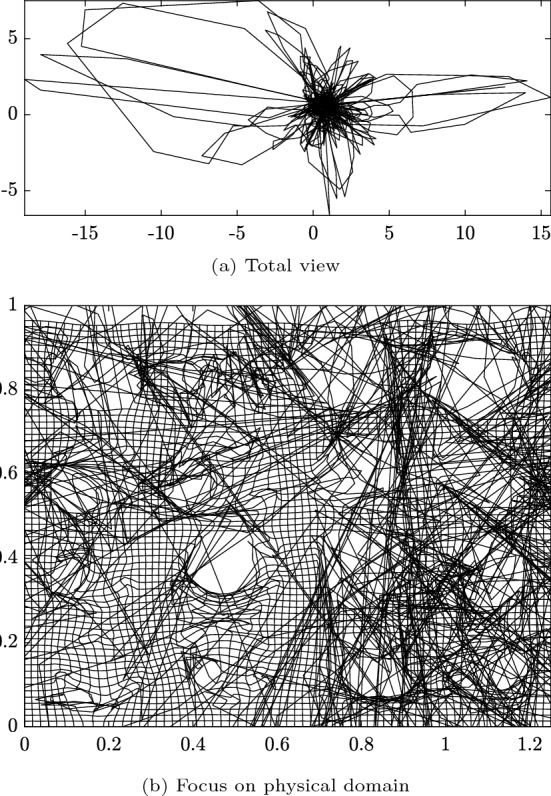
Deformed mesh for the plate with inclusions problem, including the non-physical domain. The analysis was conducted with background mesh of $64\times 64$ elements and polynomial degree of $p=2$

To illustrate the importance of deformation map resetting Fig. 21, shows the deformed configuration for one of the simulated cases. Extreme deformations can be observed within the fictitious domain. However, due to the use of deformation map resetting, such large fictitious deformations do not influence the final results. To highlight the impact of this approach, we attempted to solve the same problem without applying deformation map resetting. In this case, the solver only converged for a penalization parameter of $\alpha = 10^{-5}$. For smaller values of $\alpha$, the solver failed to converge. This experiment confirms the necessity and robustness of the deformation map resetting in maintaining numerical stability for large deformation problems. Moreover, in cases where convergence was achieved without deformation map resetting, a noticeable degradation in solution accuracy was observed. This reinforces the necessity of deformation map resetting for achieving both stable and accurate solutions in immersed finite deformation simulations.

## Conclusions

In this work, we have developed and investigated an immersed isogeometric analysis approach with boundary conformal quadrature for small and large deformation elasticity problems.

The boundary conformal quadrature points are obtained using a re-parameterization of trimmed elements using B-spline patches. This approach uses significantly fewer quadrature points than hierarchical sub-tree schemes, in particular at high polynomial orders, and is nevertheless highly accurate. This is clearly demonstrated for the linear elastic quarter plate with hole benchmark problem. Furthermore, also in the presented nonlinear elasticity examples, the desired convergence behavior is attained, which shows that the quadrature errors are insignificant. The robustness of the iterative solution of the nonlinear problems using the Newton–Raphson scheme is ensured by deformation map resetting in the fictitious domain, which prevents convergence problems in all examples studied. Furthermore, the weak enforcement of Dirichlet boundary conditions was carried out using Nitsche’s method for linear and the penalty method for nonlinear problems. In all numerical test cases, stable and accurate results were obtained using these approaches. The final numerical application for the homogenization of a microstructure RVE with pores showed the straightforward applicability of the IBC-IGA for nonlinear multiscale simulations. The results underscored the stability of the method even under significant deformations.

Our overarching conclusion is that immersed IGA with boundary conformal quadrature exhibits provides a accurate, efficient, stable, and robust numerical solution technique for complex 2D geometries also for nonlinear elasticity. Next steps will include the extension of the approach to 3D geometries, where the boundary conformal quadrature point algorithm can also be applied [4], as well as the application of the method to multiscale homogenization and simulation for real microstructures, e.g., in the context of 3D printing, and the further extension to multiphysical problems.

## Data Availability

No datasets were generated or analyzed during the current study.

## Footnotes

1 For computing those intersections we use the irit library [21] that allows to compute them up to the desired precision. Note that most CAD kernels limit the maximum precision achievable in such operations, e.g., the open source kernel Open CASCADE [60] limits the maximum precision of such operations to $10^{-9}$, what would preclude the level of accuracy reached in the convergence analyses included in Sect. 4.
